# Complete genome sequence of bacteriophage Godfather isolated from *Microbacterium foliorum*

**DOI:** 10.1128/mra.00888-24

**Published:** 2025-01-16

**Authors:** Joshua Hutchings, Lauren Bower, Ethan Collum, Timothy Hester, Melody Hunter, Chaney Kelly, Luke Reynolds, Cole Moore, Dustin Edwards

**Affiliations:** 1 Department of Biological Sciences, Tarleton State University 7612 , Stephenville, Texas, USA; Portland State University, Portland, Oregon, USA

**Keywords:** bacteriophage genetics

## Abstract

Microbacteriophage Godfather was collected from a soil sample in Stephenville, Texas. The 17,452-bp double-stranded genome contains 24 protein-coding genes. The genome shares >99% nucleotide sequence identity with cluster EE microbacteriophages Scamander, Danno, Kojax4, and Burgy.

## ANNOUNCEMENT

**Fig 1:**
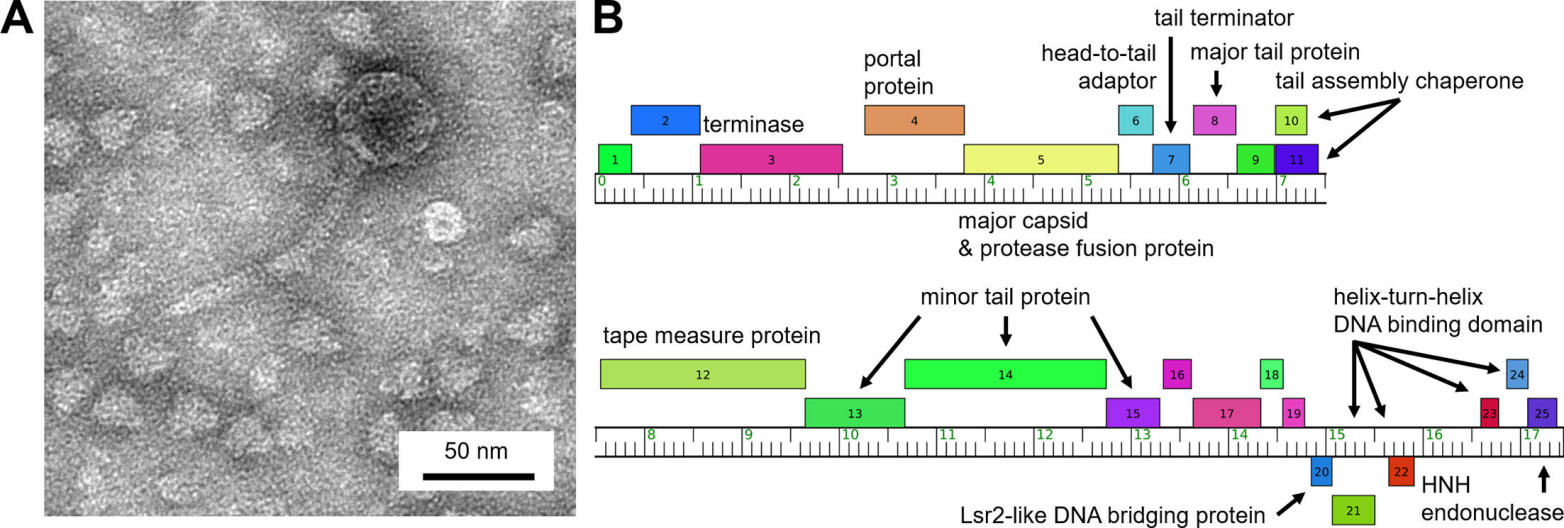
Bacteriophage Godfather virion and genome organization. (**A**) A representative micrograph of bacteriophage Godfather demonstrating siphovirus morphology with an approximate tail length of 115 nm and a capsid diameter of 50 nm. (**B**) Genes represented by boxes located above or below the ruler (forward or reverse orientation) and labeled with putative functions. Auto-annotation was performed by Starterator, Glimmer, and GeneMark, followed by manual refinement with Phamerator, PECAAN, and DNA Master. Protein-coding genes were identified by queries to BLASTp and HHPred.

Investigating the genetic diversity of bacteriophages increases our understanding of their evolution and biological roles (1–3). We isolated the bacteriophage Godfather using *Microbacterium foliorum* NRRL B-24224, which was selected as the host bacteria for its lack of prophages and internal defense systems (4, 5). A soil sample was collected from a flower bed in Stephenville, Texas (GPS coordinates 32.21191 N, 98.211755 W) and incubated with peptone yeast calcium (PYCa) media at 29°C in a shaking incubator for 2 hours (6). The sample was centrifuged at 10,000 × *g* for 20 minutes, and the supernatant was collected and filtered (0.22 µL pore size), inoculated with a freshly saturated *M. foliorum* culture, and incubated at 29°C on a PYCa plate in a soft agar overlay (6). At 24 hours, Godfather formed 2 mm clear plaques, and the bacteriophage was isolated by two rounds of ten-fold serial dilutions and plating before being prepared as a high-titer lysate (6). A 300-mesh copper grid was prepared with high-titer lysate and stained with 1% uranyl acetate. Transmission electron microscopy, using an FEI Tecnai G2 Spirit BioTWIN (NL1.160G), showed that Godfather has siphovirus morphology (Fig. 1A). ImageJ v1.53m (7) was used to measure an approximate tail length of 115 nm and a capsid diameter of 50 nm (*n* = 6).

Genomic DNA was extracted from high-titer lysate by ZnCl_2_ precipitation (8). A library was prepared with the NEBNext Ultra II kit and sequenced with an Illumina MiSeq instrument at the Pittsburgh Bacteriophage Institute (9) to 38× coverage from 30,304 single-end reads of 150 base read length. Raw reads were verified for accuracy using Consed v29.0 (9) and assembled as a single contig using Newbler v2.9 (10). The Godfather genome is 17,452 bp in length with a 9-nucleotide 3′ sticky overhang (5′-C CCGCCCCA-3′) and contains 68.7% G+C content, similar to host *M. foliorum* (5).

BLASTn (11) query of the sequence with the GenBank nonredundant/nucleotide (nr/nt) database returned nucleotide sequence identity (NSI) >99% with cluster EE bacteriophages Scamander (NC_054431), Danno (MT316462), Kojax4 (OR159657), and Burgy (ON755188) and 98% NSI to microbacteriophages Eightball (OK040783) and TimoTea (MK524502) (12–15). Of interest, Godfather shared 97% NSI with cluster EE bacteriophage Loca, which was previously isolated from a shopping cart handle at a nearby vendor that sold commercially available bagged soil (16). Starterator v546 (http://phages.wustl.edu/starterator/), Glimmer v3.02 (17), and GeneMark v2.5 (18) were used for auto-annotation, followed by manual refinement with Phamerator (19), PECAAN (https://discover.kbrinsgd.org), and DNA Master v5.23.6 (http://phagesdb.org/DNAMaster/). TmHmm v1.0.24 (20) identified potential transmembrane helices. Consistent with other cluster EE bacteriophages, no tRNA genes were identified using Aragorn v1.2.38 (21) and tRNAscan-SE v2.0 (22). Nineteen of 24 protein-coding genes were called using queries to BLASTp v2.14.1 (11) and HHPred v.3.0beta (PDB, UniProt, Pfam-A v36, and NCBI v3.19 databases) (23). Default settings were used for all programs. Functional assignments include structural proteins, an Lsr2-like DNA-bridging protein, four helix-turn-helix DNA-binding domains, and an HNH endonuclease (Fig. 1B).

## Data Availability

Godfather genome sequence is available at GenBank under the accession number PP725414 and in the SRA under accession number SRX24892095.

## References

[1] Dion MB, Oechslin F, Moineau S. 2020. Phage diversity, genomics and phylogeny. Nat Rev Microbiol 18:125–138. doi:10.1038/s41579-019-0311-5 32015529

[2] Hatfull GF. 2020. Actinobacteriophages: genomics, dynamics, and applications. Annu Rev Virol 7:37–61. doi:10.1146/annurev-virology-122019-070009 32991269 PMC8010332

[3] Jansson JK, Wu R. 2023. Soil viral diversity, ecology and climate change. Nat Rev Microbiol 21:296–311. doi:10.1038/s41579-022-00811-z 36352025

[4] Behrendt U, Ulrich A, Schumann P. 2001. Description of Microbacterium foliorum sp. nov. and Microbacterium phyllosphaerae sp. nov., isolated from the phyllosphere of grasses and the surface litter after mulching the sward, and reclassification of Aureobacterium resistens (Funke et al. 1998) as Microbacterium resistens comb. nov. Int J Syst Evol Microbiol 51:1267–1276. doi:10.1099/00207713-51-4-1267 11491322

[5] Russell DA, Garlena RA, Hatfull GF. 2019. Complete genome sequence of Microbacterium foliorum NRRL B-24224, a host for bacteriophage discovery. Microbiol Resour Announc 8:e01467-18. doi:10.1128/MRA.01467-18 30714032 PMC6357638

[6] Poxleitner M, Pope W, Jacobs-Sera D, Sivanathan V, Hatfull G. 2018. Phage discovery guide. Howard Hughes Medical Institute, Chevy Chase, MD.

[7] Schneider CA, Rasband WS, Eliceiri KW. 2012. NIH Image to ImageJ: 25 years of image analysis. Nat Methods 9:671–675. doi:10.1038/nmeth.2089 22930834 PMC5554542

[8] Santos MA. 1991. An improved method for the small scale preparation of bacteriophage DNA based on phage precipitation by zinc chloride. Nucleic Acids Res 19:5442. doi:10.1093/nar/19.19.5442 1656393 PMC328918

[9] Russell DA. 2018. Sequencing, assembling, and finishing complete bacteriophage genomes. Methods Mol Biol 1681:109–125. doi:10.1007/978-1-4939-7343-9_9 29134591

[10] Margulies M, Egholm M, Altman WE, Attiya S, Bader JS, Bemben LA, Berka J, Braverman MS, Chen Y-J, Chen Z, et al.. 2005. Genome sequencing in microfabricated high-density picolitre reactors. Nat New Biol 437:376–380. doi:10.1038/nature03959 PMC1464427 16056220

[11] Altschul SF, Gish W, Miller W, Myers EW, Lipman DJ. 1990. Basic local alignment search tool. J Mol Biol 215:403–410. doi:10.1016/S0022-2836(05)80360-2 2231712

[12] Markov SA, Church JC, Lee L, Bell CM, Binkley SD, Bouma KM, Hutson KM, Markov GS, Mason EC, Rueff GB, Sennuga TO, Simpson MH, Zimmer RJ, Villalpando DG. 2021. Complete genome sequences of Microbacterium bacteriophages Danno, Otwor, and Scumberland, isolated in Clarksville, Tennessee. Microbiol Resour Announc 10:e00209-21. doi:10.1128/MRA.00209-21 33795346 PMC8104054

[13] Van Roekel HF, Georgen JJ, Kock RA, Coleman ST. 2022. Genome sequence and characteristics of the Microbacterium foliorum cluster EE bacteriophage Burgy. Microbiol Resour Announc 11:e0091222. doi:10.1128/mra.00912-22 36197284 PMC9670927

[14] Mastropaolo MD, Fallest-Strobl PC, Sequira DM, Campbell DD, Negro CJ, Tuang KS, Sigmund-Hamre IM, Womack CL, Mansbridge T, Metzler AL, Sasher EG, Collins C, Crowley NK, Dower VR, Bates M, Bjorkelo C, Johnson H, Salvitti LR, Neumann University Phage Discovery Group. 2023. Genome sequences of five Microbacterium foliorum phages, GaeCeo, NeumannU, Eightball, Chivey, and Hiddenleaf. Microbiol Resour Announc 12:e0110622. doi:10.1128/mra.01106-22 36861977 PMC10112241

[15] Jacobs-Sera D, Abad LA, Alvey RM, Anders KR, Aull HG, Bhalla SS, Blumer LS, Bollivar DW, Bonilla JA, Butela KA, et al.. 2020. Genomic diversity of bacteriophages infecting Microbacterium spp. PLoS ONE 15:e0234636. doi:10.1371/journal.pone.0234636 32555720 PMC7302621

[16] Ounsinegad A, Ashcraft M, Bliss E, Brawley D, Clements G, Densmore A, Gastin A, Luciano M, Moore C, Munoz V, Pernarelli A, Pitner M, Velsen E, Wiggam K, Goppert M, Edwards D. 2022. Complete genome sequence of bacteriophage loca, isolated on a Microbacterium foliorum culture. Microbiol Resour Announc 11:e0078322. doi:10.1128/mra.00783-22 36066260 PMC9584219

[17] Delcher AL, Harmon D, Kasif S, White O, Salzberg SL. 1999. Improved microbial gene identification with GLIMMER. Nucleic Acids Res 27:4636–4641. doi:10.1093/nar/27.23.4636 10556321 PMC148753

[18] Besemer J, Borodovsky M. 2005. GeneMark: web software for gene finding in prokaryotes, eukaryotes and viruses. Nucleic Acids Res 33:W451–4. doi:10.1093/nar/gki487 15980510 PMC1160247

[19] Cresawn SG, Bogel M, Day N, Jacobs-Sera D, Hendrix RW, Hatfull GF. 2011. Phamerator: a bioinformatic tool for comparative bacteriophage genomics. BMC Bioinformatics 12:395. doi:10.1186/1471-2105-12-395 21991981 PMC3233612

[20] Möller S, Croning MD, Apweiler R. 2001. Evaluation of methods for the prediction of membrane spanning regions. Bioinformatics 17:646–653. doi:10.1093/bioinformatics/17.7.646 11448883

[21] Laslett D, Canback B. 2004. ARAGORN, a program to detect tRNA genes and tmRNA genes in nucleotide sequences. Nucleic Acids Res 32:11–16. doi:10.1093/nar/gkh152 14704338 PMC373265

[22] Lowe TM, Eddy SR. 1997. tRNAscan-SE: a program for improved detection of transfer RNA genes in genomic sequence. Nucleic Acids Res 25:955–964. doi:10.1093/nar/25.5.955 9023104 PMC146525

[23] Söding J, Biegert A, Lupas AN. 2005. The HHpred interactive server for protein homology detection and structure prediction. Nucleic Acids Res 33:W244–8. doi:10.1093/nar/gki408 15980461 PMC1160169

